# Frequencies of molecular markers of drug resistance in the context of two different Seasonal Malaria Chemoprevention (SMC) treatment regimens in the Koulikoro health district, Mali

**DOI:** 10.1128/aac.01806-24

**Published:** 2025-08-18

**Authors:** Bourama Traore, Fousseyni Kane, Mahamoudou Toure, Marie Helene Munck Jørgensen, Daouda Sanogo, Soumba Keita, Moussa Keita, Nafomon Sogoba, Kathryn Shaw-Saliba, Mahamadou Diakite, Jeffrey G. Shaffer, Michael Alifrangis, Helle Hansson, Seydou Doumbia

**Affiliations:** 1 University Clinical Research Center, University of Sciences, Techniques and Technologies of Bamako 225803 , Bamako, Mali; 2 Infectious Diseases and Medical Entomology Research and Training Center, University of Sciences, Techniques and Technologies of Bamako 225803 , Bamako, Mali; 3 Centre for Translational Medicine and Parasitology, Department of Immunology and Microbiology, University of Copenhagen 4321, Copenhagen, Denmark; 4 Collaborative Clinical Research Branch, Division of Clinical Research, National Institute of Allergy and Infectious Diseases, National Institutes of Health 2511, Bethesda, Maryland, USA; 5 School of Public Health and Tropical Medicine, Tulane University 25812 , New Orleans, Louisiana, USA; 6 Department of Infectious Diseases, Copenhagen University Hospital (Rigshospitalet), Copenhagen University Hospital (Rigshospitalet) 53167, Copenhagen, Denmark; Johns Hopkins University School of Medicine, Baltimore, Maryland, USA

**Keywords:** *Plasmodium falciparum*, Seasonal Malaria Chemoprevention, sulfadoxine-pyrimethamine, amodiaquine, dihydroartemisinin-piperaquine, Mali

## Abstract

With growing concern about parasite resistance to sulfadoxine-pyrimethamine (SP) in West Africa, the effectiveness of dihydroartemisinin-piperaquine (DHA + PQ) was assessed as an alternative drug regimen for Seasonal Malaria Chemoprevention (SMC). This study aims to determine the prevalence of molecular markers of resistance to SP + AQ and DHA + PQ in Koulikoro (Mali), where SMC has been implemented since 2014. *Plasmodium falciparum*-positive samples were analyzed by either next-generation sequencing, focusing on SNPs in genes known to be associated with resistance: *Pfmdr1, PfK13, Pfcrt, Pfdhfr, Pfdhps,* and *Pfexo* genes, and using qPCR for copy number variations (CNVs) of *Pfmdr1* and *Pfplasmepsin2*. A total of 564 PCR-positive *P. falciparum* samples were analyzed: 218 in 2019 and 346 in 2020. In both years, the *Pfdhfr* **51I-59R-108N** haplotype was highly prevalent (>93%). In both arms, the *Pfdhps* single mutant (**437G**) was the most prevalent (~60%). In 2020, the combined haplotype *Pfdhfr/Pfdhps* **IRN/**IA**G**KA**S** and **IRN/V**A**G**K**GS** was detected at 9.6% and 1.2%, respectively. The *Pfmdr1* haplotype (N86-**F184**-S1034-N1042-D1246) represented more than 52.0%, and no difference was observed between the years, while a significant increase in the *Pfcrt* wild-type haplotype (C72-V73-M74-N75-K76) from 39% in 2019 to 52% in 2020 (*P* = 0.01) was observed. Some *PfK13* non-synonymous mutations were observed in both years, including Y**493H** and C**580R**. The identification of the **IRN**I**/**IA**G**KA**S** quintuple mutant, along with emerging *Pfdhps***-431V** and non-synonymous PfK13-**Y493H** variants, underscores the importance of continued surveillance of resistance markers.

## INTRODUCTION

Mali is among the top 10 countries with the highest number of malaria cases and deaths: 3.1% of global cases and 3.3% of global deaths from malaria in 2021 were reported from Mali (1). The World Health Organization (WHO) global technical strategy for Malaria 2016–2030 recommended Seasonal Malaria Chemoprevention (SMC) for *P. falciparum* malaria control in children under the age of 5 years in areas with highly seasonal malaria transmission (2). The main antimalarial drug combination recommended for SMC is sulfadoxine-pyrimethamine +amodiaquine (SP + AQ) given at monthly intervals just before and during the malaria transmission season (2). SMC has been shown to prevent approximately 75% of clinical malaria episodes (3), including severe malaria. The strategy has been implemented in 15 West African countries, including Mali, since 2012 (4–6). While still impacting children under five, school-aged children now face a higher risk of symptomatic malaria (7). In 2017–2018, Mali’s Kita and Bafoulabé Districts piloted SMC for children aged 5–10 years, revealing promising outcomes for extending SMC to children aged 5–10 years, with a significant decline in malaria prevalence (8).

Resistance of *Plasmodium falciparum* to antimalarial drugs is primarily driven by single-nucleotide polymorphisms (SNPs) in parasite genes. These mutations can lead to amino acid changes that either alter the drug’s molecular target or affect drug transport and metabolism, thereby reducing drug efficacy. For SP, SNPs causing amino acid substitutions in *Pfdhps* codons I431V, S436A/F, A437G, K540E, A581G, and A613S are associated with sulfadoxine resistance, while mutations at *Pfdhfr* codons N51, C59, S108, and I164 confer pyrimethamine resistance (9). The *Pfdhps*-581G mutation, when present in combination with other mutations, forms the sextuple mutant associated with clinical failure and loss of protective efficacy of SP (10, 11). Although the impact of the 431V mutation on SP resistance remains unclear, recent studies have shown an increase in prevalence of a combination of 581G and 431V in West Africa, which warrants further investigation (12–15). For AQ, specific SNPs in *Pfmdr1*, including N86Y, F184Y, and D1246Y, as well as mutant alleles of *Pfcrt*, particularly those involving substitutions at codons C72, V73, M74, N75, and K76 (e.g., K76T), have been associated with reduced susceptibility to amodiaquine (16–18). For artemisinins, the main molecular marker of resistance is various SNPs in the *PfK13* gene (19, 20). In addition, mentioned SNPs in the *Pfmdr1* and in *Pfcrt* are associated with various levels of *P. falciparum* susceptibility to artesunate (AS) +amodiaquine (AQ) and AS +sulfadoxine-pyrimethamine (SP) (21–23).

Regarding *P. falciparum* resistance to piperaquine, a SNP (resulting in an E415G change) in *P. falciparum* exonuclease (*Pfexo*) has been associated with piperaquine resistance in Cambodia (24). In addition to *Pfexo*, mutations in the *Pfcrt* gene, such as F145I, T93S, and H97Y, have also been implicated in piperaquine resistance in Southeast Asia. However, these mutations remain rare or unreported in West Africa, and their relevance in Mali is currently unknown (25, 26). Finally, copy number variation has also been found to play a significant role in the development of antimalarial drug resistance. One copy of *Pfmdr1* is associated with slower clearance of parasites after piperaquine treatment as compared to more copies of *Pfmdr1* (27)*,* while having two copies of *P. falciparum plasmepsin2* (*Pfpm2*) is associated with recrudescent infections (25, 28).

This study aimed to determine the prevalence of molecular markers of drug resistance to sulfadoxine-pyrimethamine and amodiaquine and an alternative drug dihydroartemisinin-piperaquine across two seasons of malaria transmission (2019 and 2020) in the health district of Koulikoro, Mali, where SMC using SP + AQ has been implemented since 2012. The study was conducted in a context of a large trial to determine the effectiveness of SMC using DHA + PQ as an alternative drug compared with SP + AQ through the International Centers of Excellence in Malaria Research (ICEMR), in collaboration with Mali’s National Malaria Control Program (NMCP).

## MATERIALS AND METHODS

### Study area and sampling

**Fig 1:**
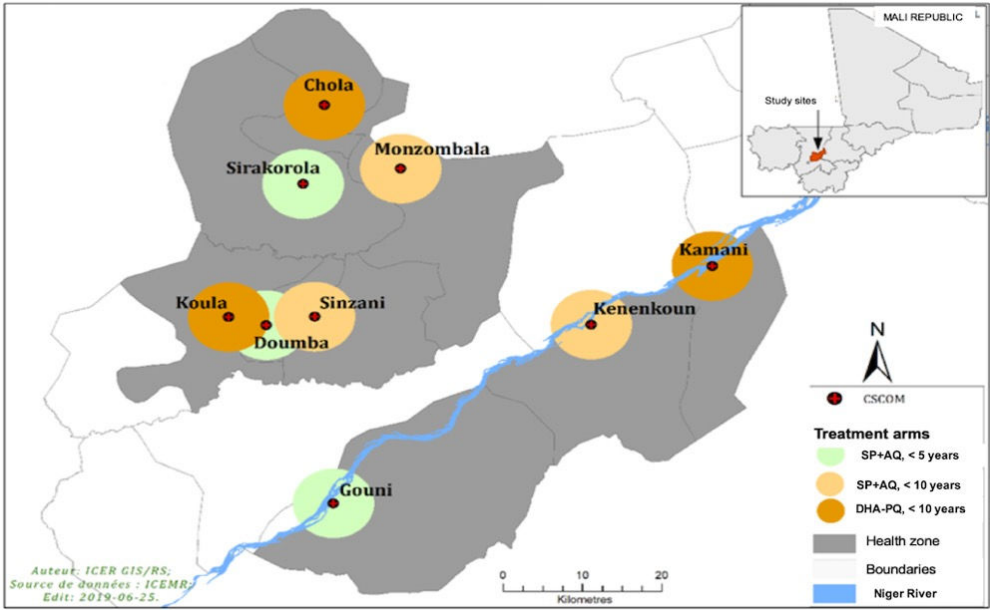
Map of Koulikoro Health District showing the study sites. Note. Map of the nine study sites in Mali’s Koulikoro District. The nine study sites are near the central part of Mali’s Koulikoro District. Study sites were selected as three villages near the Niger River, three villages in the northern part of Koulikoro District, and three villages in the central part of Koulikoro. AQ: amodiaquine; DHA: dihydroartemisinin; PQ: piperaquine; SP: sulfadoxine-pyrimethamine.

The samples were collected from the study that assessed the effectiveness of SMC using the two regimens in nine villages in the health district of Koulikoro, located in southwestern Mali (29, 30). Villages were stratified by ecological zones to ensure the sample represented the diverse malaria transmission contexts within the Koulikoro health district: along the river Niger, Central zone, and Northern zone (Fig. 1). Parental consent was obtained for all participants, and assent was sought from children aged 6 to 10 years. The study sites were randomized into three arms: Arm 1: Standard SMC with SP-AQ was delivered only to children less than 5 years old; Arm 2: SMC with SP-AQ was delivered with an age extension (up to 9 years old); and Arm 3: SMC with DHA-PQ was delivered to children up to 9 years old.

The parent study involved 1,785 participants per arm (5,355 participants overall) based on the ability to detect at least a 10% difference in malaria incidence between the comparison groups (25). Within each village, a list of eligible children was compiled, and participants were randomly selected through a proportional sampling based on the size of the eligible population, considering the relative population sizes across villages. All selected participants were tested for asymptomatic *P. falciparum* infection using microscopy. For the antimalarial drug resistance marker study, the sample size was estimated, considering a prevalence of 0.01% ± 0.1% (31), a design effect of 2, and a 95% confidence level, the minimum sample size required was 759, adjusted to 830, accounting for 10% loss due to missing or uninterpretable samples (OpenEpi) (Table S6). A total of 260 samples confirmed *P. falciparum*-positive by microscopy, and 709 samples were selected from the total pool of positive samples (~1,100), respectively, in 2019 and 2020. Positive samples with missing filter papers or incomplete blood spots were excluded. The increase in the number of positive samples in 2020 is related to the increase in the number of children screened for parasitemia compared to 2019 to ensure statistical power to detect a 10% difference between parent study arms (29, 30).

### SMC administration and sample collection

The SMC provision was performed in yearly SMC campaigns from July to October (defined as rounds). Each round consisted of four cycles, referring to the monthly administration of SP-AQ (Arm1) or DHA-PQ (Arm2) in three consecutive days (three doses per cycle). For both study arms, only the first dose was administered under directly observed therapy (DOT) by a member of the research team and the parents or guardians of the child. The second and third doses were given by parents at home according to Mali’s national SMC delivery strategy. The treatment was oral with each daily dose involving 25 mg sulfadoxine and 1.25 mg pyrimethamine per kg of body weight for SP, plus 10 mg/kg each day for AQ, and 4 mg/kg DHA and 18 mg/kg PQ for DHA-PQ. Before the initial SMC round in 2019, a unique identification number was assigned to each eligible participant to facilitate their identification throughout the study. For this study, the sampling was carried out during three cycles in 2019 (from August to October) and during four cycles in 2020 (from July to October). During each cycle, before SMC drug provision, the participants were clinically examined, and a blood smear and blood spots on filter paper were prepared for microscopy and PCR to capture the baseline genetic profiles of the parasites. During each cycle, all participants with malaria symptoms (fever) or who reported fever in the last 48 hours were immediately tested by RDT and treated according to national guidelines. To assess the frequency of molecular markers of resistance to SP +AQ and DHA-PQ, the filter paper samples were collected each year based on the positive result of microscopy, and no participant was selected twice in the same season. All RDT-positive participants, including those without symptoms, were referred for antimalarial treatment at the nearest health facility in line with national guidelines.

### Sequencing of drug resistance profiles

DNA was extracted with the E.Z.N.A. Blood DNA Mini Kit (OMEGA Bio-tek, Inc., Doraville, GA), following the manufacturer’s instructions. Briefly, a half circle 12 mm in diameter (~60 to 75 µL of blood) was punched out of the filter paper and incubated in TEN buffer (0.01M Tris-HCl, 0.001M EDTA, 0.1M NaCl, 0.1% SDS), followed by OB Protease buffer incubation. After the addition of BL buffer and ethanol, all volume was added to the HiBind1 DNA Mini Column, washed three times, and eluted in 50 µL of Elution Buffer. The extracted DNA was stored at −20°C until further analysis.

A nested PCR was applied for the detection of *Plasmodium* DNA in all extracted samples as described by Snounou et al. (28, 29). All samples that tested positive for *P. falciparum* were selected for further analysis. Genes associated with antimalarial drug resistance were amplified in a series of nested PCRs targeting fragments of *Pfdhfr, Pfdhps, Pfcrt, Pfmdr1,* and *PfK13* as described in Nag et al. (2017), but modified to run in simplex (32). In addition, for *Pfexo,* following primers for outer and nested PCR were validated and included in the pipeline, Fw 5′-CTTTAACGAATGGAGTCATTTAGCAGCA-3′, Rv 5′-TTTGATTGAATCATATCTCTATTCAATG-3′ (outer primers) and Fw 5’- **TCGTCGGCAGCGTCAGATGTGTATAAGAGACAG**TGTTAACATTTTGAAAAGATCAATTGA-3′, Rv 5’- **GTCTCGTGGGCTCGGAGATGTGTATAAGAGACAG**TGTTGAAATAGATAGAACACTAACAGT-3′ (nested primers including overhang for index primers marked in bold). The PCR products were subsequently indexed with unique index primer pairs, bead purified, and included in an amplicon-targeting sequencing on an Illumina MiSeq platform to determine the drug resistance marker profile for each sample. In the profiles, SNPs in codons N51, C59, S108, I164 of *Pfdhfr,* SNPs in codons I431, S436, A437, K540, A581, and A613 of *Pfdhps,* SNPs in codons C72, V73, M74, N75, and K76 of *Pfcrt,* SNPs in codons N86, Y184, S1034, N1042, and D1246 in *Pfmdr1,* a single SNP in codon F415 of *Pfexo* was used to build the profiles; however, the full fragments were sequenced and analyzed for SNPs, both known and unknown. The full *PfK13* gene was also included in the sequencing.

### Copy number variations of *Pfpm2* and *Pfmdr1*

In a subset of samples, multiplex qPCR targeting *Pfpm2* and *Pfmdr1* genes was performed to determine copy number variations (CNVs).

The qPCR was performed with a final volume of 25 µL consisting of 2 µL DNA extract from PCR-positive plates and 23 µL PCR Mastermix (2.5 µL *Pfmdr1* or *Pfpm2* primer mix, 2.5 µL *β-tubulin* primer mix, 12.5 µL TEMPase Hot Start, 1.25 µL gene *Pfmdr1* or *Pfpm2* probe, 1.25 µL *β-tubulin* probe, and 3 µL H2O). The cycling conditions for both targets were 50°C for 1 minute, 95°C for 10 minutes, and 45 cycles of 95°C for 15 seconds, and 58°C for 1 minute on an AriaMx machine (Agilent, Denmark). All samples were analyzed in triplicates. *β*-tubulin was used as a housekeeping gene. Laboratory 3D7 and Dd2 strains of *P. falciparum* were used as references with one copy of *Pfmdr1* (for 3D7) and multiple copies of *Pfpm2* (for Dd2) (33, 34).

The evaluation of copy numbers was based on 2^−ΔΔCt^ values. A 2^−ΔΔCt^ value above 1.50 means that there is more than one copy of the gene. A value between 0.50 and 1.49 means that there is one copy. For values below 0.50, the sample was considered negative.

### Data analysis

Raw sequencing data were processed using the Galaxy bioinformatics platform for initial quality control (QC), including adapter trimming and read filtering. High-quality reads were then aligned to the *Plasmodium falciparum* 3D7 reference genome using Python-based scripts. Variant calling was performed using a minimum threshold of 50 reads per amplicon to ensure robust SNP detection, and alleles were considered mixed if the minor allele frequency was ≥25%. SNPs of interest in *Pfdhfr, Pfdhps, Pfcrt, Pfmdr1, Pfexo*, and *PfK13* were used to define genotypes and haplotypes. The frequencies of haplotypes were expressed as proportions with corresponding 95% confidence intervals (*CIs*). The prevalence of resistance markers and haplotypes was compared between years (2019 vs. 2020) and treatment arms (SP-AQ and DHA-PQ) using chi-square or Fisher’s exact tests. A *P*-value < 0.05 was considered statistically significant. All analyses were performed using GraphPad Prism v9.5.0 and validated using Python’s SciPy library to ensure analytical reproducibility.

## RESULTS

### Summary of dried blood spot samples collected and monthly parasite positivity rates among children sampled at different points in the SMC campaign and years

DNA was extracted from a total of 969 blood samples collected on filter paper obtained from children under 5 (42%) and 5–9 years old (58%) for an antimalarial drug resistance markers study. In 2019, 240 *P*. *falciparum*-positive samples were analyzed, with 55.8% from children under 5 years old and 65.5% from children 5 to 9 years old. In 2020, the number of analyzed samples increased to 709 as the sample size was increased in the parent study (30), with children under 5 representing 44.7% and 55.3% for children 5–9 years old (Table 1). Females were more represented in both samples with 55.8% and 57.2% in 2019 and 2020, respectively (Table 1). Collectively, over the two sampling SMC rounds (yearly SMC campaign), 564 samples (58.2%) were positive for *P. falciparum* by PCR: 218 (86%) in 2019 and 346 (49%) in 2020. The parasite positivity rates for children under 5 and 5–9 years old at different points prior to monthly SMC provision are presented in Table S5. Briefly, the overall prevalence of malaria infection during the transmission season was slightly higher in 2020 (12.5%) compared to 2019 (11.2%) (*P* = 0.0402). Prevalence was higher among children aged 5–9 years old compared to those under 5 years in both 2019 (14.6% vs. 8.6%, *P* < 0.001) and 2020 (14.3% vs. 10.8%, *P* < 0.001). In 2019, the prevalence of *P. falciparum* infection was 10.8%, 10.3%, 12.4% and 11.2% in July, August, September, and October, respectively. For 2020, the prevalence rates were 10.1%, 14.6%, 13.8%, and 11.0% in July, August, September, and October, respectively (Table S5).

**TABLE 1:** Distribution of samples collected by sex, age group, and drug arms during the 2019 and 2020 SMC campaigns in Koulikoro, Mali

	2019 (*N* = 240)		2020 (*N* = 709)	
Sex	n	%	n	%
Male	145	55.8%	406	57.2%
Female	115	44.2%	303	42.8%
Age group				
<5 years	90	34.5%	317	44.7%
5–9 years	170	65.5%	392	55.3%
Drug arms				
SP + AQ	190	79.1%	423	59.6%
DHA + PQ	50	20.9%	286	40.4%

### The prevalence of SNPs in *Pfdhfr*, *Pfdhps, and Pfmdr1* in samples from 2019 and 2020

**Fig 2:**
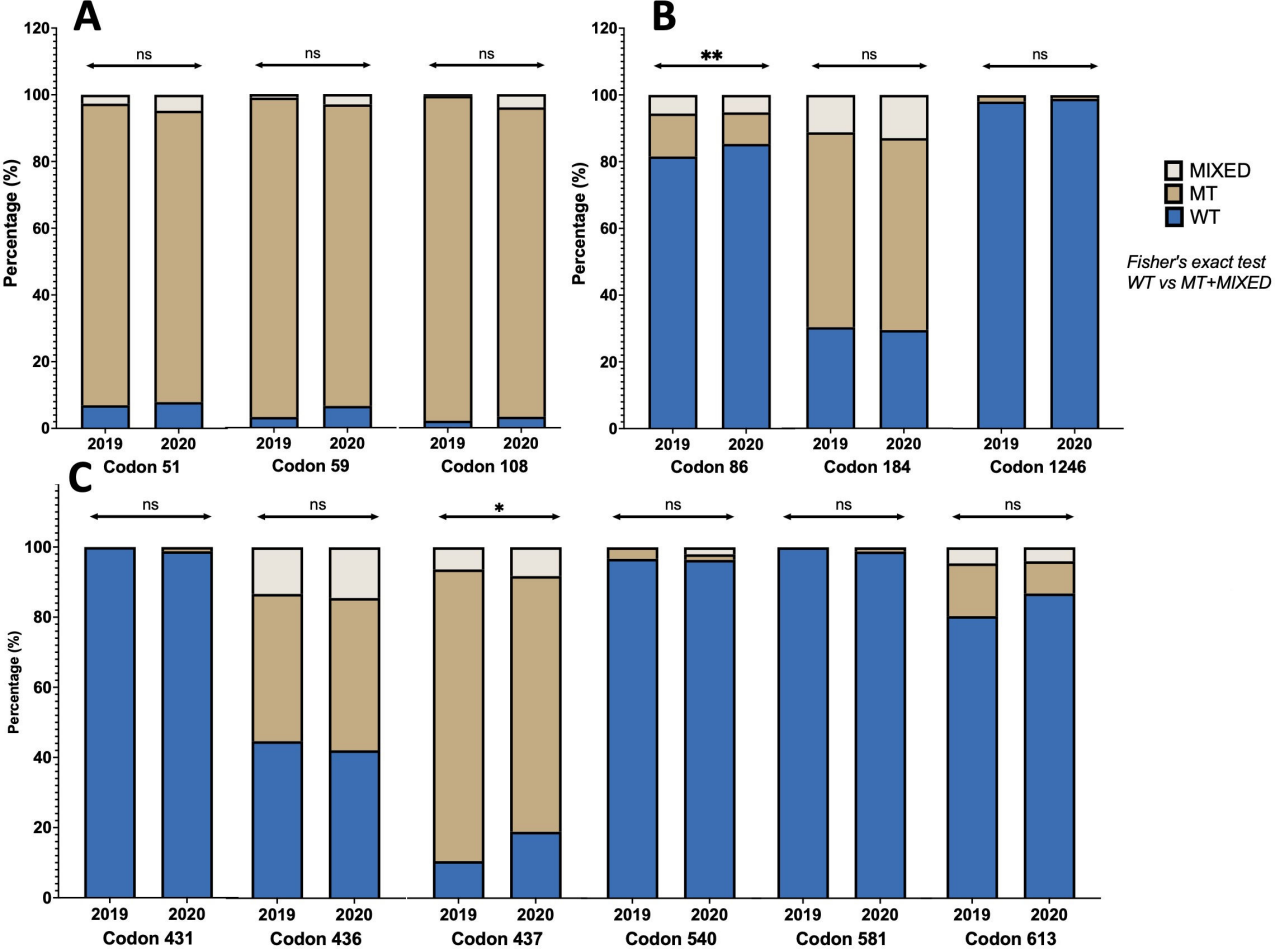
The prevalence of *P. falciparum* genotypes in samples collected in 2019 and 2020. Note. (A) *Pfdhfr* genotypes: codon 51 (wild type: N51, mutant type: I51, and mixed infections: N51/I51), codon 59 (wild type: C59, mutant type: R59, and mixed infections: C59/R59), codon 108 (wild type: S108, mutant type: N108, and mixed infections: S108/N108), and codon 164 (wild type: I164, mutant type: L164, and mixed infections: I164/L164); (B) *PfMdr1* genotypes: codon 86 (wild type: N86, mutant types: Y86 and F86, and mixed infections: N86/Y86, N86/F86), codon 184 (wild type: Y184, mutant type: F184, and mixed infections: Y184/F184), codon 1034 (wild type: S1034, mutant type: C1034, and mixed infections: S1034/C1034), codon 1042 (wild type: N1042, mutant type: D1042, and mixed infections: N1042/D1042), and codon 1246 (wild type: D1246, mutant type: Y1246, and mixed infections: D1246/Y1246). The statistical comparison between 2019 and 2020 was performed by comparing the prevalence of wild types vs. mutant type +mixed wild type/mutant type using Fisher’s exact tests; (C) *Pfdhps* genotypes: codon 431 (wild type: I431, mutant type: V431, and mixed infections: I431/V431), codon 436 (wild type: A436, mutant type: S436, and mixed infections: A436/S436), codon 437 (wild type: A437, mutant type: G437, and mixed infections: A437/G437), codon 540 (wild type: K540, mutant type: E540, and mixed infections: K540/E540), codon 581 (wild type: A581, mutant type: G581, and mixed infections: A581/G581), and codon 613 (wild type: A613, mutant type: S613, and mixed infections: A613/S613). ns = *P* ≥ 0.05,* = *P* < 0.05,** = *P* < 0.01, *** = *P* > 0.001.

All *P. falciparum-*positive samples in 2019 and 2020 based on PCR were included in the sequencing, determining genotypes and haplotypes associated with antimalarial drug resistance in codons *Pfdhfr* (51–59–108–164), *Pfdhps* (431–436–437–540–581–613), and *Pfmdr1* (86–184–1,246) shown, respectively, in Fig. 2A through C. A high prevalence of *Pfdhfr* mutants (including mixed infections) was detected at codons 51, 59, and 108, that is, 92%, 96%, and 97.8% in 2019, respectively, and again, in 2020 at 91%, 93%, and 96% (*P* > 0.05 for all comparisons of wild type versus mutant type +mixed wild type/mutant type (Fig. 2A).

The prevalence of SNPs in the different codons of *Pfdhps* for 2019 and 2020 did not show any major differences between the years (Fig. 2C) except for codon 437, where prevalence of 437G mutant type (including mixed) was 89% in 2019 versus 80% in 2020, χ^2^ = 4.71, *P =* 0.03) (Fig. 2C). No mutants were found at codons 431 and 581 in 2019; however, in 2020, 1.1% and 1.2% mutants were observed in these codons, respectively (431V, 581G) (Fig. 2C).

For *Pfmdr1* (Fig. 2B), we observed a statistically significant decrease in the prevalence of the mutant allele 86Y genotype (from 18% in 2019 to 14% in 2020 [χ^2^ = 6.70, *P =* 0.005]). For codon 184, no significant difference was observed (χ^2^ = 0.039, *P =* 0.421); the prevalence of mutant types, including mixed genotype infections, remained constant at around 70% for both years.

### The frequencies of combined *Pfdhfr-Pfdhps* haplotypes in samples from 2019 and 2020, irrespective of drug arms

Table 2 provides a comprehensive overview of combined *Pfdhf*r and *Pfdhps* haplotypes for the years 2019 and 2020, regardless of the specific drug interventions. An increase in triple mutant *Pfdhfr* haplotype (**51I-59R-108N**-I164) combined with the *Pfdhps* wild-type haplotype (I431-S436-A437-K540-A581-A613) was observed between 2019 (11/113, 9.73%; 95% confidence interval [CI] 4.42–15.93) compared to 2020 (32/166, 19.28% [95% CI 13.25–25.3], *P =* 0.0.46). The *Pfdhfr* triple mutant haplotype combined with *Pfdhps* single mutant haplotypes (I431-S436-**437G**-K540-A581-A613) was predominantly observed in both years and without apparent change, respectively (70/113, 61.9% [95% CI 53.1–70.8]) in 2019 and (96/166, 57.8% [95% CI 50.0–65.06]) in 2020 (*P =* 0.535). Conversely, the prevalence of the triple *Pfdhfr*-double *Pfdhps* (I431-S436**-437G-**K540-A581-**613S**) haplotype remained unchanged from 15.9% (95% CI 9.73–23.01) (18/113) in 2019 to 9.6% (95% CI 5.42–14.46) (16/166) in 2020 (*P =* 0.136). Finally, the triple *Pfdhfr*-double *Pfdhps* (I431-S436-**437G-540E**-A581-A613) haplotype remained low in 2019 at 3.5% (95% CI 0.88–7.08) (4/116) and in 2020 at 2.4% (95% CI 0.6–4.82) (4/166) while triple *Pfdhfr-Pfdhps*-V431 containing haplotypes were only observed in 2020, for example, **431** V-S436**-437**G-K540-A581-A613 at 0.6% (1/116) and **431** V-S436**-437**G-K540**-581G-613S** at 1.2% (2/166). Table S1 provides additional details regarding *Pfdhfr* and *Pfdhp*s haplotypes observed during 2019 and 2020 in cases where mutations are limited to three or fewer, or where there are no triple mutations in either the *Pfdhfr* or *Pfdhps* genes.

**TABLE 2:** Overview of the combined *Pfdhfr-Pfdhps* haplotypes in 2019 and 2020, irrespective of drug arms^*a*^^,^^*b*^^,^^*c*^^,^^*d*^

Number of mutations 2019	Pfdhfr	Pfdhps	Number of haplotypes				*P*-values
	Codons	Codons	2019 N	% (95 CI)	2020 N	% (95 CI)	
	51–59–108–164	431–436–437–540–581–613					
**≤3 mutations or no triple mutations in either dhfr or dhps**			8	7.08% (2.65–12.39)	15	9.04% (4.82–13.86)	0.66
2 + 2	N-**R-N**-I	I-S-**G**-K-A-**S**	2	1.77% (0.0–4.42)	–	–	0.163
3 + 0	**I-R-N**-I	I-S-A-K-A-A	11	9.73% (4.42–15.93)	32	19.28% (13.25–25.3)	**0.042**
3 + 1	**I-R-N**-I	I-S-**G**-K-A-A	70	61.95% (53.1–70.8)	96	57.83% (50.0–65.06)	0.535
3 + 2	**I-R-N**-I	I-A-**G**-K-A-**S**	18	15.93% (9.73–23.01)	16	9.64% (5.42–14.46)	0.136
3 + 2	**I-R-N**-I	**V**-S-**G**-K-A-A	**–**	–	1	0.6	*–*
3 + 2	**I-R-N**-I	I-S-**G-E**-A-A	4	3.54% (0.88–7.08)	4	2.41% (0.6–4.82)	0.718
3 + 4	**I-R-N**-I	**V**-A-**G**-K-**G-S**	**–**	–	2	1.2% (0.0–3.01)	*–*

^*a*^ Table 1 provides a comprehensive overview of combined *Pfdhfr* (*Plasmodium falciparum* dihydrofolate reductase) and *Pfdhps* (*Plasmodium falciparum* dihydropteroate synthetase) haplotypes for the years 2019 and 2020, regardless of the specific drug interventions. The table enumerates the number of mutations and corresponding haplotypes, presenting the data as both raw counts and percentages. In the first section of the table, haplotypes with limited mutations or no triple mutations in either the *dhfr* or *dhps* gene are detailed. Subsequently, the table delves into various haplotypes combining mutations in both the *dhfr* and *dhps* genes, providing a comprehensive breakdown of their occurrences for both study years. Notably, it distinguishes between haplotypes with different mutation patterns, such as "2+2," "3+0," "3+1," and "3+2," each specifying the mutations in the *Pfdhfr* and *Pfdhps* genes. These haplotypes are presented both in terms of raw counts and as a percentage of the total haplotypes observed for each respective year, providing valuable insights into the genetic diversity of *Plasmodium falciparum* parasites and potential implications for antimalarial drug resistance.

^*b*^ “–” indicates concatenated amino acid residues at sequential codons, forming a haplotype pattern across the gene.

^*c*^ Les acides aminés en gras indiquent les résidus mutants ; les acides aminés non en gras indiquent les résidus de type sauvage.

^*d*^ Bold amino acids indicate mutant residues; non-bold indicate wild-type residues.

### The prevalence of *Pfmdr1* and *Pfcrt* haplotypes in samples from 2019 and 2020, irrespective of drug arms

**Fig 3:**
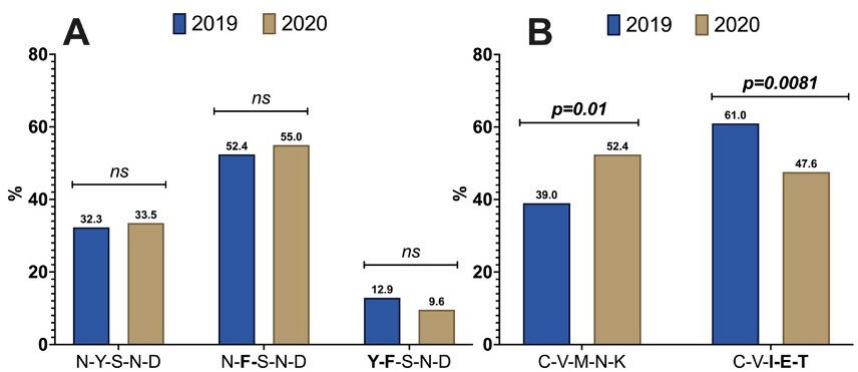
Overview of the Pfmdr1 and Pfcrt haplotypes in 2019 and 2020. Note: This bar chart provides an overview of the Pfmdr1 and Pfcrt haplotypes in 2019 and 2020. Pfmdr1 Haplotypes (Labeled “A”): We have compared the distribution of the following haplotypes: • N86-Y184-S1034-N1042-D1246: Represents the haplotype “N-Y-S-N-D.” • N86-F184-S1034-N1042-D1246: Represents the haplotype “N-F-S-N-D.” • Y86-F184-S1034-N1042-D1246: Represents the haplotype “Y-F-S-N-D.” The distribution of these haplotypes remained consistent across both years. Pfcrt Haplotypes (Labeled “B”): We compared the distribution of the following haplotypes: • C72-V73-I74-E75-T76: Represents the mutant haplotype “C-V-I-E-T.” • C72-V73-M74-N75-K76: Represents the wild-type haplotype “C-V-M-N-K.” The comparison highlights the changes in the prevalence of these haplotypes between 2019 and 2020. ns = *P* ≥ 0.05,* = *P* < 0.05,** = *P* < 0.01,*** = *P *< 0.001.

Among the different *Pfmdr1* haplotypes, no significant difference between the distribution of the three haplotypes (N86-**184F**-S1034-N1042-D1246, N86-Y184-S1034-N1042-D1246, and **86Y-184F**-S1034-N1042-D1246) was observed between 2019 and 2020 (Fig. 3A) (χ^2^ = 2.36, *P =* 0.500). For *Pfcrt* haplotypes at codons 72–76; the two haplotypes, CV**IET** and CVMNK, were observed with frequencies at 61.0% in 2019 versus 47.6% in 2020 (CVIET, χ^2^ = 7.006, *P =* 0.0081) and conversely, 39.0% in 2019 versus 52.4% in 2020 (CVMNK, χ^2^ = 7.00, *P =* 0.017) (Fig. 3B).

### Analysis of full *PfK13* gene sequence for known and unknown SNPs in 2019 and 2020

The full *PfK13* gene was sequenced and analyzed for known and unknown SNPs. Within the propeller region, non-synonymous mutations were observed: 8 in 2019 and 11 in 2020, including the WHO validated marker of partial artemisinin resistance Y493H. Another codon of interest, 580, was only found with a change in amino acids; however, not the validated marker 580Y. Only one mutation was observed more than once (W565Stop), and none of the mutations were present in both years (Table 3).

**TABLE 3:** Analysis of full *PfK13* gene sequence for known and unknown SNPs in 2019 and 2020^*a*^^,^^*b*^^,^^*c*^^,^^*d*^

SMC 2019			SMC 2020		
Codon	AA	N	Codon	AA	N
E462G	EG	1	C447Y	CY	1
G484E	GE	1	Q468STOP	STOP	1
S522C*	C	1	N499D	ND	1
V555A	VA	1	T535M	TM	1
W565STOP*	W/STOP	2	A578I	I	1
S576L	L	1	A578S*	S	1
C580R	CR	1	Y493H	YH	1
E668G	EG	1	V589I*	I	1
–	–	–	T593A	TA	1
–	–	–	G665C	C	1
–	–	–	T677I	TI	1

^*a*^ Table 2 presents an analysis of the full *PfK13* gene sequence for both known and previously unidentified SNPs observed in the years 2019 and 2020, in the context of SMC administration. For the year 2019, the table highlights specific codons within the *PfK13* gene, their corresponding amino acid (AA) changes, and the number of occurrences of each mutation. Similarly, the data for the year 2020 are presented in parallel, allowing for a comparative analysis.

^*b*^ Y493H is a validated WHO marker of artemisinin partial resistance.

^*c*^ *Mutation observed in African isolates but not associated with validated artemisinin resistance.

^*d*^ “–” indicates that no mutation was detected at the specified codon for the respective year.

Outside the propeller region, several non-synonymous mutations were found: 13 amino acid (aa) changes in codons in SMC 2019, and 27 in SMC 2020 (Table S2). Most prevalent was a mutation in codon 189, resulting in a K189T aa change. The 189T was found at 47.6% (*n* = 81/170), whereas the mixed K189T was found at 21.2% (*n* = 36/170) in 2019. In 2020, 189T were found at 45.5% (*n* = 116/255) and the K189T mixed at 20.8% (*n* = 53/255).

### Frequency of haplotypes in *Pfdhfr, Pfdhps, Pfmdr1,* and *Pfcrt* in samples from 2020 according to drugs used for SMC

In Table 4, our investigation of haplotype frequencies within *Pfdhfr, Pfdhps, Pfmdr1*, and *Pfcrt* genes among individuals receiving different antimalarial treatment arms in 2020, we observed several noteworthy trends. The **51I-59R-108N**-I164 haplotype combined with I431-S436-A437-G540-K540-A581-A613 was, respectively, observed in 22 (19.2%, [95% CI 14.14–30.3]) individuals in the SP + AQ group and 15 (22.4%, [95% CI 13.43–32.84]) individuals in the DHA + PQ group (*P >* 0.999). The triple mutant **51I-59R-108N**-I164 haplotype combined with the triple mutant *Pfdhps* **431 V**-S436-G437-K54**0-581G-613S** haplotype was not found in the SP + AQ group but was present in 2 (3%, [95% CI 0.0–7.46]) individuals in the DHA + PQ group (Table S3).

**TABLE 4:** Haplotype frequencies in 2020 for *Pfdhfr-Pfdhps* genes, and *Pfmdr1* and *Pfcrt* genes in different treatment arms^*a*^^,^^*b*^^,^^*c*^

Gene/codons	Haplotype	SP + *N* = 99 N (%, [95 CI])	DHA + *N* = 67 N (%, [95 CI])	*P*-value
	*grouped	8 (8.1%, [3.03–14.14])	7 (10.4%, [4.48–17.91])	0.595
***DHFR*** Codons 51–59–108−164/***DHPS*** Codons 431–436–437–540–581–613	**I-R-N**-I/I-S-A-K-A-A	22 (19.2%, [14.14–30.3])	15 (22.4%, [13.43–32.84])	>0.999
	**I-R-N**-I/I-**A**-A-K-A-A	17 (17.2%, [10.1–25.25])	10 (14.9%, [7.46–23.88])	0.831
	**I-R-N**-I/I-S-A-K-A-**S**	10 (10.1%, [5.05–16.16])	6 (9%, [2.99–16.42])	0.806
	**I-R-N-**I/I-S-**G**-K-A-A	40 (40.4%, [31.31–50.51])	24 (35.8%, [23.88–47.76])	0.627
	**I-R-N**-I/**V**-A-G-K-A-A	–	1(1.5%, [0.0–4.48])	*–*
	**I-R-N**-I/I-S-**G-E**-A-A	2 (2%, [0.0–5.05])	2 (3%, [0.0–7.46])	>0.999
	**I-R-N**-I/**V**-A-G-K-**G-S**	–	2 (3%, [0.0–7.46])	*–*
***MDR1*** codons 86–184–1,034–1,042–1,246	N-**F**-S-N-D	52 (50.5%, [40.78–60.19])	63 (60.6%, [50.96–70.19])	0.563
	N-Y-S-N-D	38 (36.9%, [28.16–46.6])	32 (30.8%, [22.12–39.42])	0.919
	**Y-F**-S-N-D	12 (11.7%, [5.83–18.45])	8 (7.7%, [2.88–13.46])	0.246
	**Y**-Y-S-N-**Y**	1 (1%, [0.0–2.91])	1 (1%, [0.0–2.88])	>0.999
***CRT*** codons 72–73–74–75–76	C-V-**I-E-T**	56 (45.2%, [44.66–64.08])	61 (50%, [49.04–68.27])	0.523
	C-V-M-N-K	68 (54.8%, [(56.31–74.76])	61 (50%, [49.04–68.27])	0.523

^*a*^ This table presents the frequencies of genetic haplotypes associated with antimalarial drug resistance for the year 2020, categorized by treatment arms: SP+AQ (sulfadoxine-pyrimethamine + amodiaquine) and DHA + PQ (dihydroartemisinin-piperaquine). The table includes haplotypes from *DHFR, DHPS, MDR1*, and *CRT* genes.

^*b*^ The dash (–) indicates concatenated amino acid residues at sequential codons, forming a haplotype pattern across the gene.

^*c*^ Bold amino acids indicate mutant residues; non-bold indicate wild-type residues.

For *Pfmdr1*, we found no overall differences in haplotype frequencies between the two drug arms. Specifically, the N86-**184F**-S1034-N1042-D1246 haplotype exhibited a slightly higher frequency in the DHA-PQ group (63 individuals, 60.6%, [50.96–70.19]) compared to the SP-AQ group (52 individuals, 50.5%, [40.78–60.19]). However, this difference did not reach statistical significance (*P* = 0.563).

In the *Pfcrt* gene, responsible for chloroquine resistance, we observed no significant variation in haplotype distribution based on treatment arms. Both the codons 72–76 CV**IET** and CVMNK haplotypes displayed consistent frequencies between the SP + AQ and DHA + PQ groups, with 56 individuals (45.2%, [44.66–64.08]) in the SP + AQ group and 61 individuals (50%) in the DHA + PQ group carrying the CV**IET** haplotype, while 68 individuals (54.8%, [56.31–74.76]) in the SP + AQ group and 61 individuals (50% [49.04–68.27]) in the DHA + PQ group carried the C72-V73-M74-N75-K76 haplotype.

### Prevalence of copy number variations in *Pfpm2* in samples from 2019 and 2020 and *Pfmdr1* in samples from 2020

Copy number variations (CNVs) were determined for *Pfpm2* in a subset of samples collected in 2019 (*n* = 194) and 2020 (*n* = 302). Only two samples in each sampling round were found to have more than one copy, and conversely, no differences were found between the years. CNVs were also determined for *Pfmdr1* in 134 samples collected in 2020, only. No samples were found with more than one copy (Table S4).

## DISCUSSION

The global endeavor to control and eventually eliminate malaria faces significant challenges, not least due to the emergence of drug-resistant strains of *Plasmodium falciparum*. The data presented in this study offer a comprehensive insight into the evolving landscape of molecular markers of drug resistance in *P. falciparum* populations of Mali and the potential implications for malaria control strategies using antimalarial drugs.

This study specifically assessed the variations in the frequency of molecular markers associated with antimalarial drug resistance in the context of several years of implementation of SMC in the Koulikoro health district, Mali.

SMC, widely implemented primarily in West Africa, faces increasing concerns regarding resistance to sulfadoxine-pyrimethamine (SP), evidenced by recent reports highlighting rising frequencies of resistance markers in West African populations (35). This threat of emerging resistance to SP underscores the need for evaluation of alternative regimens. Dihydroartemisinin-piperaquine (DHA-PQ) is a potential alternative with several advantages: (i) Piperaquine (PQ) is a long-acting antimalarial, making it suitable for chemoprevention and (ii) compared with SP-AQ, DHA-PQ is better tolerated and associated with lower selection of *dhfr* and *dhps* mutations (36). While DHA-PQ represents a promising alternative to SP-AQ for SMC in areas of rising SP resistance, concern about the risk of accelerating resistance emergence has been raised with its widespread use, as observed in Southeast Asia (25). Thus, evaluating the effect of these drug regimens on the prevalence and frequency of resistance-associated molecular markers is essential to guide the implementation of SMC.

Our results revealed that the prevalence of molecular markers associated with resistance to SP-AQ and DHA-PQ did not vary significantly over the 2 years (2019 and 2020) and did not compromise the use of either regimen. No mutations associated with piperaquine resistance—such as those in Pfcrt or increased Pfpm2 copy number—were detected in this study (24, 37). The *Pfexo* E415G mutation, which has been reported in Southeast Asia in association with piperaquine treatment failures, was not observed (38).

Overall, there were no significant differences in the prevalence of SNPs in *Pfdhfr* and *Pfdhps* between 2019 and 2020, except for codon 437**G** of *Pfdhps*. Our study did not identify any increase in the prevalence of specific *Pfdhfr-Pfdhps* haplotypes, including the quintuple mutant haplotype associated with drug resistance, consistent with recent findings from Mali and other West African countries (37, 39, 40). This suggests that the prevalence of these genotypes remained stable during the study period, supporting the continued effectiveness of SMC in reducing malaria incidence (31, 41–43). A slight increase in the prevalence of triple *Pfdhfr* haplotype **I51-R59-N108**-I164 combined with the *Pfdhps* wild-type haplotype was observed from 2019 to 2020.

Regarding the *Pfdhps* 431V mutation, despite the low prevalence observed (1.2%), these results align with previous studies, including Benin in 2021 (3.9%), Mali in 2014 (increase from 0.2% to 3.2%), and Nigeria between 2003 and 2017 (increase from 0% to 51%) (13, 14, 44, 45). The potential detrimental impact of V431-containing haplotypes on preventive strategies such as SMC and IPTp remains unclear. However, their emergence warrants careful monitoring, as it might represent a developing West African equivalent of the East African sextuple *Pfdhfr-Pfdhps* haplotype known to compromise the chemoprevention effectiveness (46, 47). Importantly, despite the presence of molecular markers of resistance to SP, no particular selection was observed, supporting the continued use of SMC as an effective malaria intervention in the region (35, 46, 48).

For *Pfmdr1*, a significant decrease in the prevalence of the codon Y86 mutant (including mixed N86/86Y infections) was observed from 18% in 2019 to 14% in 2020, raising important questions about the evolving drug resistance dynamics (49). The predominance of wild-type *Pfmdr1* and *Pfcrt* haplotypes observed in this study may be influenced by the national treatment policy favoring artemether-lumefantrine as first-line therapy. Lumefantrine has been associated with the selection of wild-type alleles at codons N86 and K76 in several settings, and this pressure may contribute to maintaining these genotypes in the parasite population (35). The observed decrease in the prevalence of this *Pfmdr1* mutant indicates the absence of SMC-induced selection, a hypothesis further strengthened by the lack of difference between the distribution of the three haplotypes N86-**F184**-S1034-N1042-D1246, N86-Y184-S1034-N1042-D1246, and **Y86-F184**-S1034-N1042-D1246 observed between 2019 and 2020, and between the different drug groups.

While the two SMC regimens exert distinct drug selection pressures, our comparison of molecular marker prevalence across arms was descriptive in nature and not intended to imply direct causality (SMC using SP + AQ has been implemented in the study area since 2014). Nonetheless, such contrasts provide valuable baseline insights into resistance dynamics under different chemoprevention strategies and support ongoing molecular surveillance to guide SMC policy.

Regarding mutations in the *PfK13* gene, the WHO-validated partial artemisinin resistance marker Y493H was detected in a single mixed infection sample (50). This marker may confer ART resistance *in vitro* or at least delayed parasite clearance (39, 51–53). Although this mutation is more common in Southeast Asia, its identification in West Africa highlights the importance of ongoing surveillance to detect and respond to such occurrences rapidly. While other *PfK13* mutations associated with artemisinin resistance have been documented in Tanzania, Uganda, the DRC, and southern Rwanda (54–58), to the best of our knowledge, we report for the first time the mutation Y493H in this region.

Other mutations identified in the propeller region (A578S, W565Stop, S522C, V589I, and C580R) have not been associated with resistance *in vitro*. Although A578S is relatively common, it was only found once in this study (in SMC 2020) (59). Outside the propeller region, the K189T mutation was found to be highly prevalent. This is similar to other studies, but no association with delayed clearance or resistance has been found (60).

Our investigation into copy number variations (CNVs) in *Pfmdr1* and *Pfpm2* found no evidence of increases associated with piperaquine resistance (61), suggesting that the SMC program has not resulted in an increased prevalence of CNVs associated with resistance to the drugs used in this study (62).

A limitation of our study is the relatively short duration of the 2-year observation period, which may not be sufficient to detect significant changes in the prevalence of molecular markers of antimalarial drug resistance. However, this study provides a strong baseline data on the distribution of molecular markers of antimalarial drug resistance in the Koulikoro region and guides evidence-based deployment for continuing with SP-AQ or alternative drug regimen for SMC. Longer-term monitoring and larger sample sizes will be necessary to further elucidate the impact of SMC on drug resistance in the study area.

### Conclusion

In conclusion, our findings suggest that the prevalence of molecular markers of antimalarial drugs does not compromise the SMC strategy using SP + AQ or alternative drug of DHA-PQ in the Koulikoro health district of Mali. However, the finding for the first time in the region of the WHO-validated *PfK13* marker Y493H, a rare mutation in Africa, highlights the importance of molecular monitoring to detect and address potential spread of artemisinin resistance and its implications for malaria control strategies in the region.

## Data Availability

All sequencing data generated and analyzed during this study are publicly available in the NCBI Sequence Read Archive (SRA) under the BioProject accession number PRJNA1299543 (https://www.ncbi.nlm.nih.gov/sra/PRJNA1299543). Clinical metadata and individual-level information associated with the samples have been de-identified in accordance with ethical guidelines and are not included in the public repository. However, additional metadata may be made available upon reasonable request to the corresponding author, subject to approval by the Ethics Committee of the University Clinical Research Center (UCRC), University of Sciences, Techniques and Technologies of Bamako (USTTB), Mali.

## SUPPLEMENTAL MATERIAL

Table S1 aac.01806-24-s0001.docx Genetic haplotypes (≤3 mutations or no triple mutations) in 2019 and 2020: *Pfdhfr* and *Pfdhps* mutations irrespective of drug arms.

Table S2 aac.01806-24-s0002.docx *Pfk13* Mutations outside the propeller region.

Table S3 aac.01806-24-s0003.docx Summary of *Pfdhfr* and *Pfdhps* haplotypes (≤3 mutations or no triple mutations) in 2020 by antimalarial treatment arms.

Table S4 aac.01806-24-s0004.docx *Plasmepsin2* and *Pfmdr1* copy number variations per years.

Table S5 aac.01806-24-s0005.docx Monthly prevalence of *Plasmodium falciparum* infection among children aged <5 and 5–9 years in 2019 and 2020.

Table S6 aac.01806-24-s0006.docx Sample size for frequency in a population.

ASM does not own the copyrights to Supplemental Material that may be linked to, or accessed through, an article. The authors have granted ASM a non-exclusive, world-wide license to publish the Supplemental Material files. Please contact the corresponding author directly for reuse.
