# Supplementary material for: Frequencies of molecular markers of drug resistance in the context of two different Seasonal Malaria Chemoprevention (SMC) treatment regimens in the Koulikoro health district, Mali
Source: Antimicrob Agents Chemother. 2025 Aug 18;69(10):e01806-24. doi: 10.1128/aac.01806-24 (PMC12486799; doi:10.1128/aac.01806-24)
Supplement: Table S1 — Genetic haplotypes (≤3 mutations or no triple mutations) in 2019 and 2020: Pfdhfr and Pfdhps mutations irrespective of drug arms. [file aac.01806-24-s0001.docx]

**Supplementary table1: Genetic Haplotypes (≤3 mutations or no triple mutations) in 2019 and 2020: *Pfdhfr* and *Pfdhps* Mutations Irrespective of Drug Arms**

| ≤3 mutations or no triple mutations in either dhfr or dhps*  **Number of mutations** | ***PfDHFR*** | ***PfDHPS*** | **Number of haplotypes 2019** | **%** | **Number of haplotypes 2020** | **%** |
| --- | --- | --- | --- | --- | --- | --- |
|  | **Codons 51-59-108-164** | **Codons 431-436-437-540-581-613** |  |  |  |  |
| 0+0 | N-C-S-I | I-A-A-K-A-A | 1 | 0,88 | 4 | 2,41 |
| 0+1 | N-C-S-I | I-S-**G**-K-A-A |  |  | 4 | 2,41 |
| 0+2 | N-C-S-I | I-A-**G**-K-A-**S** | 1 | 0,88 |  |  |
| 1+0 | N-C-**N**-I | I-A-A-K-A-A | 1 | 0,88 |  |  |
| 1+1 | N-C-**N**-I | I-S-**G**-K-A-A | 1 | 0,88 |  |  |
| 2+0 | N-**R**-**N**-I | I-S-A-K-A-A | 1 | 0,88 |  |  |
| 2+0 | N-**R**-**N**-I | I-A-A-K-A-A | 1 | 0,88 | 1 | 0,60 |
| 2+1 | N-**R**-**N**-I | I-S-**G**-K-A-A | 1 | 0,88 | 2 | 1,20 |
| 2+1 | **I**-C-**N**-I | I-S-**G**-K-A-A |  |  | 1 | 0,60 |
| 2+1 | **I**-C-**N**-I | I-A-**G**-K-A-A |  |  | 1 | 0,60 |
| 2+1 | N-**R**-**N**-I | I-A-**G**-K-A-A | 1 | 0,88 | 2 | 1,20 |

**Note. –** The supplemental table 1 provides additional details regarding Pfdhfr (*Plasmodium falciparum* dihydrofolate reductase) and Pfdhps (*Plasmodium falciparum* dihydropteroate synthetase) haplotypes observed during 2019 and 2020 in cases where mutations are limited to three or fewer, or where there are no triple mutations in either the *dhfr* or *dhps* genes. This specific subset of haplotypes is of interest due to its potential significance in antimalarial drug resistance. The table includes the number of mutations within the *Pfdhfr* and *Pfdhps* genes, highlighting various mutation patterns represented by haplotypes such as "0+0," "0+1," "0+2," "1+0," "1+1," "2+0," and "2+1," each indicating mutations at specific codons. The frequency of these haplotypes is presented both for the year 2019 and the year 2020, expressed as raw counts and percentages of the total haplotypes identified.This supplemental table offers a more detailed breakdown of specific haplotype compositions within the context of limited mutations or absence of triple mutations in the dhfr and dhps genes contributing to a comprehensive understanding of the genetic diversity of Plasmodium falciparum parasites in the study population.
