# Supplementary material for: Frequencies of molecular markers of drug resistance in the context of two different Seasonal Malaria Chemoprevention (SMC) treatment regimens in the Koulikoro health district, Mali
Source: Antimicrob Agents Chemother. 2025 Aug 18;69(10):e01806-24. doi: 10.1128/aac.01806-24 (PMC12486799; doi:10.1128/aac.01806-24)
Supplement: Table S2 — Pfk13 Mutations outside the propeller region. [file aac.01806-24-s0002.docx]

**Supplementary table2: *Pfk13* Mutations outside the propeller region**

| SMC 2019 | | | SMC 2020 | | |
| --- | --- | --- | --- | --- | --- |
| Codon | **AA** | **N** | **Codon** | **AA** | **N** |
| S37N | SN | 1 | L62I | LI | 1 |
| P96S | S | 1 | D93N | DN | 1 |
| M106I | I | 1 | R101G | G | 1 |
| G112E | E | 1 |  | RG | 2 |
|  | GE | 1 | K108E | KE | 2 |
| N136C | C | 1 | I111R | IR | 1 |
| T149S | TS | 2 | G112E | E | 1 |
| A175T | T | 1 |  | GE | 4 |
| K189T |  |  | K124R | KR | 1 |
|  | T | 81 | T149S | TS | 1 |
|  | KT | 36 | S161L | L | 1 |
| K189N | N | 5 | L177I | LI | 1 |
|  | KN | 2 | K189T |  |  |
| K217Q | Q | 1 |  | T | 116 |
| R255K | K | 3 |  | KT | 53 |
|  | RK | 7 | K189N | N | 9 |
| R308G | RG | 1 |  | KN | 7 |
| E426G | EG | 1 | K217Q | KQ | 1 |
|  |  |  | D221V | DV | 1 |
|  |  |  | T225I | TI | 1 |
|  |  |  | I227T | IT | 2 |
|  |  |  | R255K | K | 2 |
|  |  |  |  | RK | 3 |
|  |  |  | L258M | M | 1 |
|  |  |  |  | LM | 1 |
|  |  |  | E261G | EG | 1 |
|  |  |  | R265H | RH | 1 |
|  |  |  | N345D | ND | 1 |
|  |  |  | V356A | VA | 1 |
|  |  |  | S381N | SN | 1 |
|  |  |  | F409P | FP | 1 |
|  |  |  | R411K | K | 1 |
|  |  |  | S425N | SN | 1 |
|  |  |  | G436D | GD | 1 |

**Note. –** Comparison of Mutations Outside the Propeller Region in SMC (Seasonal Malaria Chemoprevention) Samples from 2019 and 2020.

- SMC 2019: Number of occurrences of specific codon mutations and corresponding amino acid changes

SMC 2020: Number of occurrences of specific codon mutations and corresponding amino acid changes
