# Supplementary material for: Frequencies of molecular markers of drug resistance in the context of two different Seasonal Malaria Chemoprevention (SMC) treatment regimens in the Koulikoro health district, Mali
Source: Antimicrob Agents Chemother. 2025 Aug 18;69(10):e01806-24. doi: 10.1128/aac.01806-24 (PMC12486799; doi:10.1128/aac.01806-24)
Supplement: Table S3 — Summary of Pfdhfr and Pfdhps haplotypes (≤3 mutations or no triple mutations) in 2020 by antimalarial treatment arms. [file aac.01806-24-s0003.docx]

**Supplementary table 3: Summary of *Pfdhfr* and *Pfdhps* Haplotypes (≤3 mutations or no triple mutations) in 2020 by Antimalarial Treatment Arms**

| ≤3 mutations or no triple mutations in either dhfr or dhps* | ***PfDHFR*  Codons 51-59-108-164** | ***PfDHPS*  Codons 431-436-437-540-581-613** | **SP+AQ** | **DHA+PQ** |
| --- | --- | --- | --- | --- |
| **0+1** | N-C-S-I | I-A-A-K-A-A | 4 | - |
| **0+1** | N-C-S-I | I-S-G-K-A-A | 2 | 2 |
| **2+0** | **I**-C-**N**-I | I-A-G-K-A-A | 1 | - |
| **2+0** | N-**R-N**-I | I-A-G-K-A-A | - | 2 |
| **2+1** | **I**-C-**N**-I | I-S-**G**-K-A-A | 1 | - |
| **2+1** | N-**R-N**-I | I-A-**A**-K-A-A | - | 1 |
| **2+1** | N-**R-N**-I | I-S-**G**-K-A-A | - | 2 |

**Note. –** This table provides a summary of haplotypes associated with drug resistance markers in 2020 for samples subjected to different antimalarial treatment arms: SP+AQ (Sulfadoxine-Pyrimethamine + Amodiaquine) and DHA+PQ (Dihydroartemisinin-Piperaquine). The table specifically focuses on haplotypes for *DHFR* (Dihydrofolate Reductase) and *DHPS* (Dihydropteroate Synthase) genes with ≤3 mutations or no triple mutations in either dhfr or dhps*.
