# Supplementary material for: Frequencies of molecular markers of drug resistance in the context of two different Seasonal Malaria Chemoprevention (SMC) treatment regimens in the Koulikoro health district, Mali
Source: Antimicrob Agents Chemother. 2025 Aug 18;69(10):e01806-24. doi: 10.1128/aac.01806-24 (PMC12486799; doi:10.1128/aac.01806-24)
Supplement: Table S4 — Plasmepsin2 and Pfmdr1 copy number variations per years. [file aac.01806-24-s0004.docx]

**Supplementary table 4: *Plasmepsin2* and Pf*mdr1* copy number variations per years.**

| **Copy Numbers** | ***Plasmepsin2*** | | ***Mdr1*** |
| --- | --- | --- | --- |
|  | **SMC 2019, N (%)** | **SMC 2020, N (%)** | **SMC 2020, N (%)** |
| 1 copy | 192 (99) | 300 (99.3) | 134 (100) |
| > 1 copy | 2 (1) | 2 (0.7) | 0 |
| Total | 194 (100) | 302 (100) | 134 (100) |

**Note. –** This table presents the copy number variations of *Plasmepsin2* and *Mdr1* genes for the years 2019 and 2020 among study participants receiving Seasonal Malaria Chemoprevention (SMC). The data illustrates the distribution of individuals with different copy numbers, with a focus on the prevalence of one copy or more than one copy for both genes.
