# Supplementary material for: Frequencies of molecular markers of drug resistance in the context of two different Seasonal Malaria Chemoprevention (SMC) treatment regimens in the Koulikoro health district, Mali
Source: Antimicrob Agents Chemother. 2025 Aug 18;69(10):e01806-24. doi: 10.1128/aac.01806-24 (PMC12486799; doi:10.1128/aac.01806-24)
Supplement: Table S5 — Monthly prevalence of Plasmodium falciparum infection among children aged <5 and 5–9 years in 2019 and 2020. [file aac.01806-24-s0005.docx]

|  |  | **Jully**  **N (%)** | **August**  **N (%)** | **Sept**  **N (%)** | **Oct**  **N (%)** | **Total**  **N (%)** | ***p-value*** |
| --- | --- | --- | --- | --- | --- | --- | --- |
| **2019** | **<5 years** | - | 711 (10.1%) | 642 (7.2%) | 586 (8.2%) | **1939 (8.6%)** | ***<0.0001*** |
|  | **5-9 years** | - | 389 (12.1%) | 525 (14.1%) | 544 (16.9%) | **1458 (14.6%)** |  |
|  | **Total** | **-** | **1100 (10.8%)** | **1167 (10.3%)** | **1130 (12.4%)** | **3397 (11.2%)** |  |
|  |  |  |  |  |  |  |  |
| **2020** | **<5 years** | 900 (8.8%) | 1230 (13.7%) | 1021 (12.4%) | 977 (7.4%) | **4128 (10.8%)** | ***<0.0001*** |
|  | **5-9 years** | 959 (11.3%) | 1194 (15.5%) | 944 (15.4%) | 911 (14.8%) | **4008 (14.3%)** |  |
|  | **Total** | **1859 (10.1%)** | **2424 (14.6%)** | **1965 (13.8%)** | **1888 (11%)** | **8136 (12.5%)** |  |

**Supplemetary table 5: Monthly Prevalence of *Plasmodium falciparum* Infection Among Children Aged <5 and 5–9 Years in 2019 and 2020.**
