# Supplementary material for: Frequencies of molecular markers of drug resistance in the context of two different Seasonal Malaria Chemoprevention (SMC) treatment regimens in the Koulikoro health district, Mali
Source: Antimicrob Agents Chemother. 2025 Aug 18;69(10):e01806-24. doi: 10.1128/aac.01806-24 (PMC12486799; doi:10.1128/aac.01806-24)
Supplement: Table S6 — Sample size for frequency in a population. [file aac.01806-24-s0006.docx]

**Supplementary table 6: Sample Size for Frequency in a Population**

Population size (for ﬁnite population correction factor or fpc)(N): ~ 30000

Hypothesized % frequency of molecular markers of antimalarial drug resistance in the population (p): 0.01%+/-0.1

Conﬁdence limits as % of 100 (absolute +/- %) (d): 0.1%

Design effect (for cluster surveys-DEFF): 2

**Sample Size(*n*) for Various Conﬁdence Levels**

| **ConﬁdenceLevel(%)** | **Sample Size** |
| --- | --- |
| 95% | 759 |
| 80% | 327 |
| 90% | 537 |
| 97% | 928 |
| 99% | 1299 |
| 99.9% | 2090 |
| 99.99% | 2883 |

**Equation**

Sample size n = [DEFF*Np(1-p)]/ [(d2/Z21-α/2*(N-1)+p*(1-p)]

Assuming a 10% of loss of samples or uninterpretable analysis (759 + 759 * 0.1), the minimum sample size was 830.

Results from OpenEpi, Version 3, open source calculator--SSPropor (<http://www.openepi.com/SampleSize/SSPropor.htm>)
